# The Value of Systemic Inflammatory Indices for Predicting Early Postoperative Complications in Colorectal Cancer

**DOI:** 10.3390/medicina60091481

**Published:** 2024-09-11

**Authors:** Irina Shevchenko, Catalin Cicerone Grigorescu, Dragos Serban, Bogdan Mihai Cristea, Laurentiu Simion, Florentina Gherghiceanu, Andreea Cristina Costea, Dan Dumitrescu, Catalin Alius, Corneliu Tudor, Minodora Onisai, Sebastian Gradinaru, Ana Maria Dascalu

**Affiliations:** 1 Faculty of Medicine, Carol Davila University of Medicine and Pharmacy Bucharest, 020021 Bucharest, Romania; irina.shevchenko@drd.umfcd.ro (I.S.); laurentiu.simion@umfcd.ro (L.S.); f.gherghiceanu@umfcd.ro (F.G.); dan.dumitrescu@umfcd.ro (D.D.); catalin.alius@umfcd.ro (C.A.); corneliu.tudor@umfcd.ro (C.T.); minodora.onisai@umfcd.ro (M.O.); ana.dascalu@umfcd.ro (A.M.D.); 2 Fourth Department of General Surgery, Emergency University Hospital Bucharest, 050098 Bucharest, Romania; 3 Faculty of Medicine, Titu Maiorescu University, 040441 Bucharest, Romania; cicerone.grigorescu@prof.utm.ro (C.C.G.); sebastian.gradinaru@prof.utm.ro (S.G.); 4 Department of Surgical Oncology, Institute of Oncology “Prof. Dr. Al. Trestioreanu”, 022328 Bucharest, Romania; 5 Department of Nephrology, Diaverum Clinic, 900612 Constanta, Romania; andreea.costea@diaverum.com; 6 Hematology Department, Emergency University Hospital Bucharest, 050098 Bucharest, Romania; 7 Department of General Surgery, Ilfov County Emergency Clinical Hospital, 022104 Bucharest, Romania

**Keywords:** colorectal cancer, complications, surgery, systemic inflammatory indices, neutrophil-to-lymphocyte ratio (NLR), systemic inflammatory index (SII), platelet-to-lymphocyte ratio (PLR)

## Abstract

*Background and Objectives*: Systemic inflammatory indices have been largely investigated for their potential predictive value in multiple inflammatory, infectious, and oncological diseases; however, their value in colorectal cancer is still a subject of research. This study investigates the dynamics of pre- and postoperative values of NLR, PLR, SII, and MLR in patients with colorectal cancer and their predictive value for early postoperative outcomes. *Materials and Methods*: A 2-year retrospective cohort study was performed on 200 patients operated for colorectal adenocarcinoma. Systemic inflammatory indices were calculated based on complete blood count preoperatively and on the first and sixth postoperative days. The patients were divided into two groups based on their emergency or elective presentation. The pre- and postoperative values of serum inflammatory biomarkers and their correlations with postoperative outcomes were separately analyzed for the two study subgroups. *Results*: There were no significant differences in sex distribution, addressability, associated comorbidities, or types of surgery between the two groups. Patients in the emergency group presented higher preoperative and postoperative values of WBC, neutrophils, NLR, and SII compared to elective patients. The postsurgery hospital stays correlated well with pre- and postoperative day one and day six values of NLR (*p* = 0.001; 0.02; and <0.001), PLR (*p* < 0.001), SII (*p* = 0.037; <0.001; <0.001), and MLR (*p* = 0.002; *p* = 0.002; <0.001). In a multivariate analysis, reintervention risk was higher for emergency presentation and anemia, and lower in right colon cancer. In the emergency group, a multivariate model including age, MLR PO1, and pTNM stage was predictive for severe postoperative complications (AUC ROC 0.818). First-day postoperative inflammatory indices correlated well with sepsis, with the best predictive value being observed for the first postoperative day NLR (AUC 0.836; sensibility 88.8%; specificity 66.7%) and SII (AUC 0.796; sensitivity 66.6%; specificity 90%). For elective patients, the first postoperative day PLR and anemia were included in a multivariate model to predict Clavien–Dindo complications graded 3 or more (AUC ROC 0.818) and reintervention (AUC ROC 0.796). *Conclusions*: Easy-to-calculate and inexpensive systemic inflammatory biomarkers could be useful in predicting early postoperative outcomes in colorectal cancer for both elective and emergency surgery.

## 1. Introduction

In recent years, colorectal cancer has consistently been ranked as the third most common cancer among all types of malignant tumors. It ranks second in terms of mortality, with an increasing trend and early onset at younger ages [1,2]. In 2018, around 1.8 million new cases and approximately 881,000 deaths related to this disease were estimated worldwide [3,4]. However, despite the efficient prevention that could be achieved by influencing modifiable risk factors, implementing screening programs, and the early removal of precancerous lesions, the incidence of colorectal cancer is expected to rise globally, reaching 3.2 million new cases and 1.6 million deaths by 2040 [1]. In Romania, the number of newly diagnosed cases increases by an average of 4000 patients each year, and the mortality rate remains unacceptably high [5,6].

Open and laparoscopic procedures are the mainstay therapy, along with chemotherapy depending on the tumoral stage. Colorectal oncological surgery is, however, associated with a significant incidence of postoperative complications, which may deeply impact patients’ survival, oncological prognosis, and quality of life [7,8]. Moreover, up to 20% of cases are admitted in emergency situations, with occlusion or perforation [9]. Surgery in these cases has been reported to be associated with a 3- to 10-fold increased rate of operative mortality and higher postoperative morbidity and mortality [9,10]. As complications are considered a marker of quality of care [8,11], there is a high demand for new biomarkers and personalized therapies to improve perioperative outcomes in patients with colorectal cancer.

Many modern problems find solutions by turning to discoveries and theories of the past. As such, inflammation indices have been widely studied by contemporary researchers in line with Virchow’s proposal in 1863 that cancer is a systemic disease that is inseparably linked to the immune system [12]. Another renowned scientist, Dvorak, shared a similar view, defining a malignant tumor as a non-healing wound [12,13].

This has led to the proposal of various formulas for calculating inflammation indices in recent years. These formulas may be useful to assess risks and predict potential early and late complications [14,15,16]. The low cost and simplicity of determining and calculating inflammatory indices have led to this method steadily securing its position in the diagnosis, the assessment of treatment effectiveness, and the prognosis assessment of patients in cardiology, oncology, rheumatology, and even endocrinology [17,18,19,20]. The most commonly mentioned indices in the literature are NLR (neutrophil-to-lymphocyte ratio), PLR (platelet-to-lymphocyte ratio), MLR (lymphocyte-to-monocyte ratio), and SII (systemic immune-inflammation index).

Multiple studies have evidenced that inflammatory indices are a reliable tool in assessing the long-term prognosis of cancer patients [21,22]. However, there is limited evidence regarding their role in the early prediction of postoperative risk in the same group of patients [23,24,25].

In the present study, we investigated the pre- and postoperative dynamics of systemic inflammatory indices and their correlation with early postoperative outcomes in patients with colorectal cancer. Taking into account the obvious differences in inflammatory and immune status, a separate statistical analysis was carried out for elective versus complicated colorectal cancer requiring emergency surgery.

## 2. Materials and Methods

### 2.1. Study Design

We conducted a retrospective cohort study between January 2022 and December 2023 on patients with colorectal cancer admitted for surgery in our department. The research was conducted according to STROBE criteria for cohort studies (Supplementary file S1). All patients aged over 18 years for whom the diagnosis of colorectal cancer could be documented by histopathological exam and who underwent open and laparoscopic surgical procedures, either in an emergency or on an elective basis, were included. Patients with a lack of necessary data were excluded.

To avoid potential bias, patients with other co-existing pathologies previously documented to impact the blood cells and influence systemic inflammatory indices, such as complicated diabetes, associated infections, and hematological and autoimmune diseases, were excluded. Moreover, taking into account the different inflammatory statuses between patients presented in emergency for complicated colorectal cancer and those presented on an elective basis, correlations between systemic inflammatory indices and various outcomes were analyzed separately for each subgroup.

Information on demographics, clinical and surgical details, laboratory results, hospital stays, and postoperative complications was obtained from the patients’ clinical and surgical reports. For our study, the following clinical data were extracted from medical records: gender, age, comorbidities, the patient’s general condition at the time of hospitalization according to the ASA classification, type of admission (emergency, elective), presence of complications related to the primary oncological condition (anemia due to recurrent bleeding, abscess, perforation, bowel obstruction), tumor site, type of surgical intervention, histopathological characteristics of the neoplasm (including pTNM stage), number of days of hospitalization after surgery, readmission within 30 days of discharge, postoperative complications, and laboratory data.

Comorbidities referred to the presence of other chronic diseases in the patient that were not related to their oncological condition, and were defined as follows: obesity (patients with a BMI greater than 30) anemia (defined according to our laboratory standards as hemoglobin levels less than 10.9 g/dL and/or red blood cell counts less than 3.6 × 10^6^/μL), and patients with previously diagnosed diabetes mellitus or patients with HbA1c levels above 6.5% at the time of admission. We used the Charlson Comorbidity Index (CCI) to evaluate the severity and presence of multiple comorbidities for the patients included in the study. The study was conducted according to the Declaration of Helsinki and approved by the Ethical Committee of the Emergency University Hospital Bucharest (Ethical approval no. 20.10.63568). 

Based on the type of presentation and the moment of surgery, the study group was divided into an elective group (planned surgery after comprehensive previous evaluation) and an emergency group (admission in emergency situation for complicated colorectal cancer, surgery performed within 24 h of admission). The pre-and postoperative values (means ± SD) of serum inflammatory biomarkers were comparatively analyzed in the 2 study subgroups. Furthermore, their correlations and predictive value for postoperative outcomes were analyzed separately in each specific group.

All hospitalized patients underwent preoperative preparation and postoperative monitoring according to hospital guidelines, which mandatorily include a complete blood count on admission, the first postoperative day, and the sixth postoperative day (when applicable). Informed consent for surgery was obtained in all cases after presenting the therapeutic alternative and reasonable disclosure [26]. Fasting blood samples were taken following all national and international standards, with a complete blood count. Inflammatory indices were calculated based on the counts of neutrophils, platelets, and neutrophils measured from the same sample and expressed as their value in cells/L [24,27,28]. SII was calculated by the following formula: SII = neutrophil count × platelet count/lymphocyte count [29]. Charlson Comorbidity Index (CCI) scores were calculated retrospectively for the patients enrolled in the study based on the comorbidities documented in their observation charts.

### 2.2. Statistical Analysis

The data were analyzed using Microsoft Excel, EasyMedStat (version 3.36; www.easymedstat.com. accessed at 12 August 2024), and Med Calc^®^ Statistical Software (version 22.006 Med Calc Software Ltd., Ostend, Belgium; https://www.medcalc.org; accessed on 10 August 2024). Numeric variables were expressed as means (±SD) and discrete outcomes as absolute and relative (%) frequencies. The statistical analysis between study groups was assessed by Pearson’s Chi-squared test and Fisher’s exact test for discrete variables, and ANOVA for continuous variables. A *p*-value of <0.05 was considered statistically significant. For relevant parameters, a multiple regression analysis was conducted to identify the best predictive models for postoperative complications.

## 3. Results

During the study period, 200 patients with colorectal cancer underwent surgery in our department, of whom 74 were admitted in emergency (emergency group) and 126 underwent elective procedures (elective group). The mean age of the cohort was 68 ± 10.1 years. Notably, there was a significantly higher age in the emergency vs. elective group (*p* = 0.01); see Table 1.

There were no significant differences in terms of sex distribution, addressability, or associated comorbidities between the two groups. However, the ASA risk grade at admission was higher in the emergency group, possibly due to associated complications (*p* < 0.001).

All patients requiring urgent surgery experienced complications related to their oncological condition. The most predominant was bowel obstruction (21 cases, 56.7%), with septic issues such as abscess formation, tumor perforation, and peritonitis (9 cases, 24.3%) being the second most prevalent complication. In seven (18.9%) cases, the surgery was mandatory due to inferior digestive bleeding.

In terms of biological characteristics, patients in the emergency group presented higher preoperative and postoperative values of WBC, neutrophils, NLR, and SII, compared to elective patients (Table 2). All four parameters increased postoperatively in both study groups.

Postoperatively, these indicators increased in both groups. Preoperative fibrinogen was higher in the emergency group (*p* < 0.01). However, on day 1 after surgery, the difference became non-significant due to a different dynamic of this inflammatory biomarker in the two groups. It increased in the elective group and decreased in the emergency group. Additionally, platelet counts were higher in the emergency cohort on day one after intervention (*p* < 0.01). Postoperative PLR was increased in the emergency group; however, the difference was not significant (*p* = 0.16).

### 3.1. Surgery and Postoperative Outcomes

All patients included in the study underwent resection surgery with tumor removal. Of these, 37% of patients were treated with laparoscopic surgery, while the remaining 63% underwent open surgery. The type of surgery was chosen according to tumor location and the viability of the surrounding tissues, with the most frequent surgeries being sigmoidectomy (37%), right hemicolectomy (28%), and rectal amputation (13%). There was no difference in terms of type of surgery (*p* = 0.15) or preference for laparoscopic approach between the two study groups (*p* = 0.61, Table 3). However, a temporary colostomy/ileostomy was more often used in emergency cases (62.16% vs. 34.9%, *p* = 0.01).

Postoperative hospital stay was significantly longer for patients undergoing emergency surgery compared to the elective group (14.5 days vs. 10.9 days, *p* < 0.001). Postoperative complications were documented in 67 patients and classified according to the Clavien–Dindo classification. Statistical analysis showed a significant difference in the rate and distribution of complications according to the Clavien–Dindo classification in the two study groups, with cases requiring intensive (grade IV) care and resulting in death (grade V) being mostly encountered in the emergency group (Table 3).

In our study, 22 patients required reintervention, but in only 13 cases was the surgery sufficient to resolve the postoperative complication. In the remaining cases, seven patients needed intensive care (Clavien–Dindo grade 4), and two deaths (Clavien–Dindo grade 5) were also observed. The rate of reintervention was higher in the emergency group (16.2% vs. 7.9%, *p* = 0.04). The causes that required surgery are presented in Table 4.

Most deaths were observed in the emergency group (16 cases), with the major cause being uncontrolled sepsis with multiple system organ failure (MSOF) (14 out of 16 cases, 87.5%). In the elective group, only two deaths were encountered and were caused by pneumonia with acute respiratory failure, one due to COVID-19 hospital-acquired infection.

### 3.2. Correlations between Systemic Inflammatory Indices for Postoperative Outcomes

The correlations between systemic inflammatory indices and postoperative outcomes were evaluated for each subgroup in terms of postoperative hospital stay, severe Clavien–Dindo complications (grade ≥ 3), the risk of reintervention and, for the emergency group, postoperative sepsis. A cut-off value of ≥3 was chosen for the Clavien–Dindo grade of postoperative complications, based on the fact that severe complications (those graded III, IV, or V) significantly correlate with a decrease in physical and psychological QOL during the early postoperative period [30].

#### 3.2.1. Predictive Value of Systemic Inflammatory Biomarkers in the Emergency Subgroup

In the emergency group, preoperative values of NLR, PLR, SII, and MLR did not correlate with any of the investigated postoperative outcomes.

ANOVA tests found a significant correlation between the length of postoperative hospital stay and the postoperative values of NLR (NLR PO1 *p* < 0.001; NLR PO6 *p* < 0.001), SII (SII PO1 *p* < 0.001; SII PO6 *p* < 0.001), MLR (MLR PO1 *p* = 0.03; MLR PO6 *p* < 0.001), and PLR (PLR PO6 *p* < 0.001). Overall, severe postoperative complications (Clavien–Dindo ≥ 3) correlated with higher first-day postoperative values of PLR (*p* = 0.04) and MLR (*p* = 0.005). The mean values of first-postoperative-day NLR and SII were higher in patients that experienced complications, but not statistically significant (*p* = 0.061; *p* = 0.063, respectively).

None of the studied biomarkers could be correlated with the rate of reintervention, which could be explained by the different causes that required an early surgical approach, but also the multiple factors (patient- and procedure-related) that were involved.

Higher first-day postoperative values of NLR, PLR, SII, and MLR were significantly associated with postoperative sepsis (*p* < 0.001 for all parameters). Interestingly, none of the preoperative or day 6 postoperative values were correlated with the risk of septic complications.

**Figure 1:**
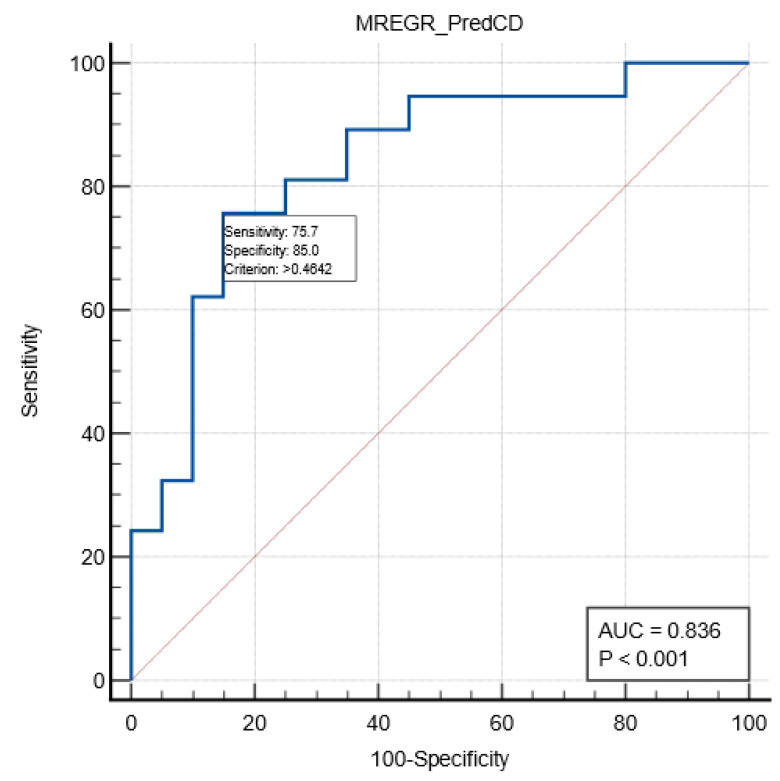
ROC curve (blue color) describing the prediction of severe complications in the emergency group by the multivariate model.

A multivariate regression analysis was performed to assess the relation between relevant variables and postoperative Clavien–Dindo complications in the emergency sub-group. A model including age, first-day postoperative MLR, and pTMN stage was described, with a good predictive value for severe complications (AUC ROC 0.836; sensitivity 75.7%; specificity 85%; cut-off value of >0.46). See Table 5 and Figure 1.

The first-day postoperative values of all investigated biomarkers (NLR, PLR, MLR, SII) were significantly correlated with postoperative sepsis. However, their predictive value assessed by ROC curves varied from low to good. Among the investigated biomarkers, the first-day postoperative NLR value had the best predictive power for sepsis at a cut-off value of >12.57, with good sensitivity (88.8%) but fair specificity (66.6%, AUC ROC 0.837).

**Figure 2:**
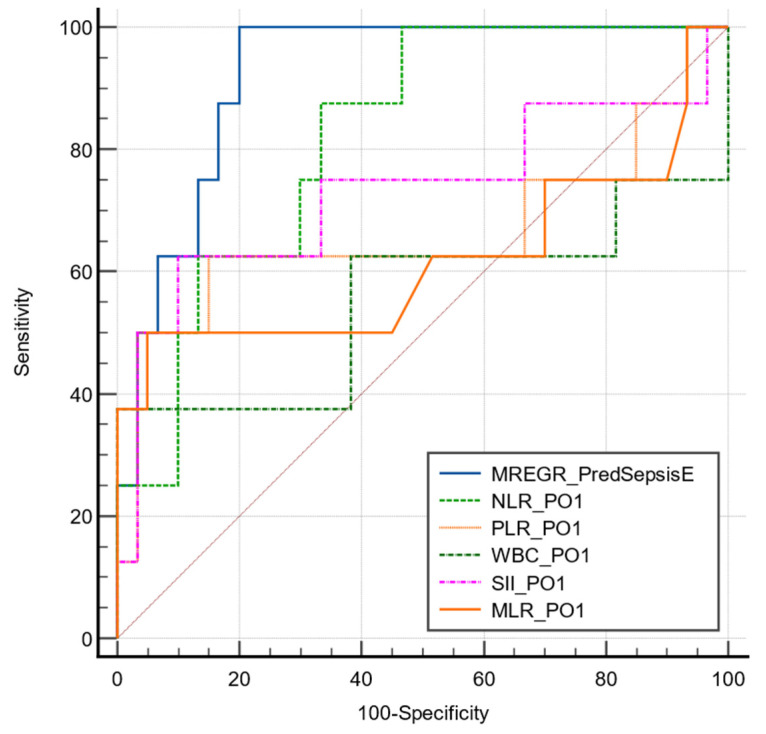
Comparative ROC curve for the described model of sepsis in emergency subgroup.

A multivariate regression analysis combining NLR PO1 and the necessity of performing colostomy/ileostomy at the initial surgery proved to have an excellent predictive value (AUC ROC 0.921, *p* < 0.001; sensitivity 80%; specificity 100%; cut-off value >0.29). See Table 6 and Figure 2.

The multivariate model presented a significantly higher predictive value compared to each biomarker alone (*p* > 0.05). The area under the ROC curve for each parameter is presented in Table 7.

#### 3.2.2. Predictive Value of Systemic Inflammatory Biomarkers in Elective Subgroup

In the elective group, the length of hospital stay correlated with postoperative NLR (*p*-values of <0.001 and <0.001, respectively), and both preoperative and postoperative values of PLR (*p*-values of 0.002; <0.001; and <0.001, respectively), MLR (*p*-values of <0.001; <0.001; and <0.001, respectively) and SII (*p*-values of <0.001; <0.001; and <0.001, respectively).

Higher preoperative and first-day postoperative values of PLR were associated with postoperative severe complications (*p* = 0.01; *p* = 0.002, respectively) and a higher rate of reintervention (*p* = 0.02; *p* = 0.002, respectively), while no correlations were observed with NLR pre- and postoperative values. Moreover, a higher preoperative MLR was associated with reintervention (*p* = 0.02), and an increased first-day postoperative SII correlated well with severe complications (*p* = 0.01).

**Figure 3:**
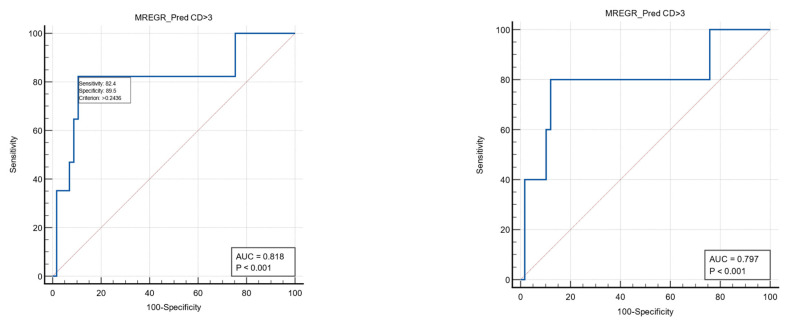
ROC curves (blue color) to describe the predictive power of the multivariate model for severe postoperative complications (**left**) and reinterventions (**right**).

The multivariate analysis found a regression model combining the first postoperative PLR value and the presence of anemia to be predictive for severe postoperative complications (Clavien–Dindo grade ≥3) with a sensitivity of 82.4%, specificity of 89.5%, and a cut-off value of >0.24 (AUC ROC: 0.818). The same model may be able to predict reinterventions but with a slightly lower value (AUC ROC: 0.797; sensitivity 80%; specificity 87.9%; associated criterion > 0.24). See Table 8 and Figure 3.

## 4. Discussion

The scientific world is structured in such a way that when a new door opens, a new direction emerges, becomes fashionable, and sets new trends. Today, this direction can confidently be considered immunology. Particularly noteworthy is the role-playing and role-switching behavior of immune cells in the context of oncological diseases, which, unfortunately, still challenge modern medicine and require increasing efforts from science and medical personnel. In addition to well-studied histopathological biomarkers predictive for long-term survival, such as MSI (microsatellite instability), KRAS, and BRAF mutations [31], current studies search for personalized approaches to enhance early postoperative outcomes and prevent perioperative morbidity [32,33]. Several studies have shown that different biological markers may be useful to identify postoperative complications early, such as CRP [34,35], Il-6, YKL-40 [35], tumor necrosis factor-α (TNF-α), and interleukin-10 (IL-10) [36]. However, tests are expensive, with limited availability in clinical practice in many countries.

Systemic inflammatory biomarkers can be easily assessed based on complete blood count, and they may provide information regarding imbalances in inflammatory status and immunological response. There are recent studies that correlate systemic inflammatory indices with postoperative morbidity and survival. Zhang et al. [37] found that preoperative SII was an independent biomarker for overall survival in gastrointestinal cancer. Cook et al. [38] found that NLR > or =9.3 on the first postoperative day is associated with an increased risk of complications, while Josse et al. found that preoperative NLR ≥ 2.3 may be a risk factor for major surgical complications following colorectal resection [39]. However, more data are needed to better understand the perioperative dynamics of these biomarkers and the levels that should be considered as indicative of risk.

In the present paper, we aimed to explore whether inflammatory indices are useful for assessing the risk of early postoperative complications to improve the quality of life of patients with colorectal cancer requiring surgery. Furthermore, preoperative and two postoperative values (at day 1 and day 6) were analyzed comparatively across the elective and emergency groups. We documented an increase in NLR, SII, PLR, and MLR on the first postoperative day in elective and emergency-operated patients, with a progressive decrease towards day 6. However, initial values were not reached. Fibrinogen presented a different dynamic in the two study groups, with significant differences being noted only in the preoperatory state. This finding may be explained by changes related to surgical stress, tissue destruction, and inflammatory reactions that may alter the hemostatic–fibrinolysis balance [40,41].

We found different correlations between the systemic inflammatory biomarkers and postoperative outcomes in the emergency versus elective surgery subgroups. We observed significantly increased NLR and SII in the emergency group compared to the elective group for both the pre- and postoperative values. Notably, NLR PO1 seems to perform better than SII PO1 in predicting sepsis, with higher accuracy (AUC 0.821 vs. 0.729), and could be a valuable tool in clinical practice. The information provided by these two biomarkers is complementary, with NLR presenting higher sensitivity (88.8% vs. 66.7%) and SII better specificity (90% vs. 66.6%). It is also important to note that patients who require surgery in an emergency are at a higher risk of developing such complications compared to those who have elective surgeries. This finding is expected but requires more attention and possibly greater efforts to prevent these adverse events [7,8,9]. Moreover, a multivariate model including age, MLR PO1, and TMN stage was described for overall severe postoperative complications, with good predictive value.

In the elective group, PLR and anemia played a more important role in predicting postoperative outcomes than NLR and SII, showing a different inflammatory and immune profile between the two study subgroups. This finding supports the need for the enhanced preoperative preparation of each patient, and the correction of metabolic and nutritional imbalances to prevent complications [42,43,44].

All studied inflammatory indices from the preoperative and postoperative tests in our study showed statistically significant correlations with length of hospitalization. However, none of them correlated with reintervention in the emergency subgroup. This fact may be explained by the relatively low level of cases with these complications, and the heterogeneity of causes that required surgical or endoscopic approaches.

In our study, 37% of patients presented in emergency situations for complicated colorectal cancer. This high proportion may be partially explained by disruptions in screening programs and delays in medical care during the COVID-19 pandemic [45,46,47].

In recent years, the behavior, ratios, and interactions of immune cells in benign and malignant pathologies have been actively studied in oncology to assess cancer stage and long-term prognosis [4,5,48]. One of the limitations of our study was that most cases were in stages 2–3, for which a correlation of NLR with tumoral stage could not be assessed. The incidence of anastomotic leak was low (3%), so the potential value of systemic inflammatory biomarkers for this dreadful complication could not be assessed. Further studies on a larger number of patients are needed to confirm a potential correlation between NLR values and this dreaded complication associated with colon and rectal surgery. Nevertheless, the data obtained from this study provide additional information and may help direct further research.

## 5. Conclusions

Systemic inflammatory biomarkers are valuable in predicting septic complications and adverse outcomes in colorectal cancer, both in emergency and elective surgery. Dynamic evaluation, based on both pre- and early postoperative data, is useful in predicting prognosis postsurgery. Additionally, this approach does not require additional costs or skills, and can be implemented in healthcare facilities with limited resources.

## Figures and Tables

**Table 1:** Demographic and general data of the patients included in the study.

Variable	Total N = 200	Elective N = 126	Emergency N = 74	*p*-Value
Age (mean ± SD, years)	68.01 ± 10.1	66.1 ± 9.1	71.27 ± 10.9	0.01 ^1^
Gender M (n, %)	98 (49%)	64 (50.8%)	34 (45.9%)	0.79 ^2^
Area (n, %)				0.4 ^2^
Rural	100 (50%)	68 (53.9%)	32 (43.2%)	
Urban	100 (50%)	59 (46.1%)	41 (56.8%)	
Charlson Comorbidity Index (n, %)				0.14 ^2^
2	32 (16%)	18 (14.3%)	14 (18.9%)	
3	78 (39%)	42 (33.3%)	36 (48.7%)	
4	52 (26%)	33 (26.1%)	19 (25.0%)	
5	27 (13.5%)	22 (17.5%)	5 (5.4%)	
6	11 (5.5%)	10 (7.9%)	1 (1.3%)	
Obesity (n, %)	31 (15.5%)	22 (17.5%)	9 (11.8%)	0.78 ^2^
DM II (n, %)	40 (20%)	28 (22.2%)	12 (16.2%)	0.6 ^2^
Anemia (n, %)	76 (38%)	50 (39.7%)	26 (35.1%)	0.81 ^2^
ASA grade (n, %)				<0.001 ^3^
1	2 (1%)	2 (1.6%)	0 (0.0%)	
2	40 (20%)	40 (31.7%)	0 (0.0%)	
3	92 (46%)	82 (65.1%)	10 (13.5%)	
4	66 (33%)	2 (1.6%)	64 (86.5%)	
Cancer site (n, %)				0.46 ^3^
Right colon	56 (28%)	30 (23.7%)	26 (35.2%)	
Left colon	11 (5.5%)	9 (7.1%)	4 (5.4%)	
Sigmoid	85 (42.5%)	52 (41.3%)	32 (43.2%)	
Rectum	48 (24%)	36 (28.6%)	12 (16.2%)	
TNM stage * (n, %)				0.07 ^4^
I	26 (13%)	12 (19.05%)	2 (2.7%)	
II	54 (27%)	17 (26.98%)	20 (27.03%)	
III	105 (52.5%)	62 (49.21%)	45 (60.4%)	
IV	15 (7.5%)	6 (4.76%)	9 (12.1%)	
Tumoral grading (n, %)				0.62 ^4^
G1	12 (6%)	8 (6.4%)	4 (5.4%)	
G2	175 (87.5%)	112 (88.8%)	63 (85.1%)	
G3	13 (6.5%)	6 (4.8%)	7 (9.4%)	

Footnote: * TNM staging was based on histopathological exam of the resection piece; ^1^ Student’s T-test (unpaired); ^2^ Chi-squared test; ^3^ two-tailed Fisher’s exact test; ^4^ Mann–Whitney test; a *p*-value of <0.05 was considered statistically significant. Abbreviations: DM II: diabetes mellitus II; ASA: American Society of Anesthesiologists’ Physical Status Classification System.

**Table 2:** Biological characteristics on admission and first postoperative day in elective and emergency groups.

Variable (Mean ± SD)	Total N = 200	Elective N = 126	Emergency N = 74	*p*-Value *
WBC (cells × 10^3^/MMC)	8.33 ± 4.72	6.98 ± 3.41	10.61 ± 5.83	<0.001
Lymphocytes (cells × 10^3^/MMC)	1.34 ± 0.71	1.29 ± 0.62	1.42 ± 0.81	0.54
Neutrophils (cells × 10^3^/MMC)	6.12 ± 4.4	4.88 ± 3.13	8.23 ± 5.57	<0.001
Monocytes (cells × 10^3^/MMC)	0.61 ± 0.33	0.57 ± 0.26	0.66 ± 0.41	0.31
Hb (mg/dL)	11.17 ± 1.87	11.34 ± 2.0	10.89 ± 1.64	0.24
PLT (cells × 10^3^/MMC)	305.28 ± 131.7	291.52 ± 128.52	328.7 ± 137.24	0.12
Fb (mg/dL)	409.68 ± 129.48	374.38 ± 86.36	469.78 ± 166.83	<0.01
AST (U/L)	48.9 ± 11.6	43.2 ± 16.2	58.9 ± 12.6	0.63
ALT (U/L)	67.2 ± 15.1	52.7 ± 19.7	75.8 ± 16.9	0.06
Creatinine (mg/dL)	1.3 ± 0.5	1.2 ± 0.3	1.5 ± 1.4	0.34
NLR	6.52 ± 8.71	4.63 ± 3.94	9.74 ± 12.92	0.02
PLR	307.58 ± 325.24	267.38 ± 154.69	376.02 ± 494.95	0.66
MLR	0.58 ± 0.57	0.52 ± 0.27	0.7 ± 0.87	0.7
SII	2161.5 ± 4213.77	1431.43 ± 1868.37	3404.58 ± 6386.63	0.004
WBC PO1 (cells × 10^3^/MMC)	11.48 ± 5.73	9.94 ± 3.68	14.11 ± 7.54	<0.001
Lymphocytes PO1 (cells × 10^3^/MMC)	0.98 ± 1.11	0.86 ± 0.43	1.17 ± 1.74	0.68
Neutrophils PO1 (cells ×10^3^/MMC)	9.69 ± 5.49	8.17 ± 3.42	12.26 ± 7.26	<0.001
Monocytes PO1 (cells × 10^3^/MMC)	0.78 ± 0.46	0.71 ± 0.3	0.89 ± 0.64	0.31
PLT PO1 (cells × 10^3^/MMC)	261.33 ± 116.9	234.27 ± 98.5	307.41 ± 133.38	<0.01
Hb PO1 (mg/dL)	10.22 ± 1.19	10.22 ± 1.24	10.22 ± 1.13	0.83
Fb PO1 (mg/dL)	410.45 ± 108.01	402.81 ± 101.01	423.46 ± 120.67	0.42
NLR PO1	14.21 ± 15.47	11.87 ± 8.93	18.19 ± 22.39	<0.01
PLR PO1	384.75 ± 336.67	336.96 ± 214.94	466.12 ± 473.8	0.19
MLR PO1	1.09 ± 0.94	0.97 ± 0.52	1.29 ± 1.4	0.6
SII PO 1	3713.39 ± 3705.43	2774.15 ± 2250.38	5312.63 ± 5024.47	<0.01
WBC PO6 (cells × 10^3^/MMC)	8.96 ± 4.3	7.73 (±3.25)	11.12 (±5.14)	<0.001
Lymphocytes PO6 (cells × 10^3^/MMC)	1.29 ± 1.52	1.34 (±1.88)	1.19 (±0.531)	0.105
Neutrophils PO6 (cells × 10^3^/MMC)	7.62 ± 8.84	5.71 (±2.94)	10.96 (±13.7)	<0.001
Monocytes PO6 (cells × 10^3^/MMC)	0.63 ± 0.25	0.594 (±0.251)	0.7 (±0.252)	0.013
PLT PO6 (cells × 10^3^/MMC)	272.62 ± 116.37	257.57 (±110.0)	298.94 (±125.49)	0.036
NLR PO6	8.27 ± 8.26	6.63 (±4.3)	11.15 (±12.12)	0.087
PLR PO6	303.38 ± 199.5	294.75 (±158.58)	318.49 (±260.09)	0.471
MLR PO6	0.70 ± 0.44	0.68 (±0.38)	0.729 (±0.535)	0.965
SII PO6	2400.68 ± 3552.77	1690.82 (±1361.13)	3642.94 (±5470.51)	0.02
FbPO6	436.9 ± 135.4	496.5 (±133.26)	497.62 (±143.88)	0.563

Footnote: * calculated by Mann–Whitney test. Abbreviations: WBC: white blood cells; Hb: hemoglobin; PLT: platelets; Fb: fibrinogen; PO1: postoperative day one; NLR: neutrophil-to-lymphocyte ratio; PLR: platelet-to-lymphocyte ratio; MLR: lymphocyte-to-monocyte ratio; SII: systemic immune-inflammation index; PO6: postoperative day 6.

**Table 3:** Surgical treatment and postoperative outcomes.

Variable	Total N = 200	Elective N = 126	Emergency N = 74	*p*-Value *
Surgical approach (n, %)				0.61 ^1^
Open	126 (63%)	76 (60.32%)	50 (67.57%)	
Laparoscopic	74 (37%)	50 (39.68%)	24 (32.43%)	
Type of surgery (n, %)				0.15 ^1^
Right hemicolectomy	56 (28%)	30 (23.81%)	26 (35.14%)	
Left hemicolectomy	6 (3%)	2 (1.59%)	4 (5.41%)	
Sigmoid segmentary resection	1 (0.5%)	1 (0.8%)	0 (0.0%)	
Sigmoidectomy	75 (37.5%)	44 (34.92%)	31 (41.8%)	
Total colectomy	1 (0.5%)	0 (0.0%)	1 (1.35%)	
Rectal amputation	26 (13%)	12 (9.52%)	4 (5.41%)	
Recto sigmoidectomy	25 (12.5%)	24 (19.05%)	1 (2.7%)	
Anterior rectal resection	18 (9%)	12 (9.6%)	6 (8.11%)	
Colostomy/ileostomy (n, %)	89 (44.5%)	43 (34.1%)	46 (62.16%)	0.01 ^1^
Postoperative hospital stays (days, mean ± SD)	12.28 ± 7.72	10.92 ± 7.81	14.59 ± 7.19	<0.001 ^2^
Readmission within 30 days (n, %)	5 (2.5%)	2 (1.59%)	3 (4%)	0.55 ^1^
Overall complications (n, %)	67 (33.5%)	25 (19.8%)	42 (56.7%)	<0.001 ^1^
Clavien–Dindo classification for postoperative complications * (n, %)				<0.001 ^2^
I (surgical-site infection)	13 (6.5%)	8 (6.35%)	5 (6.7%)	
II (requiring pharmacological treatment)	8 (4%)	6 (4.76%)	2 (2.7%)	
III (requiring endoscopic/surgical/Rx approach)	13 (6.5%)	8 (6.35%)	5 (6.7%)	
IV (requiring intensive care)	15 (7.5%)	2 (3.2%)	13 (16.22%)	
V (death)	18 (9%)	2 (1.58%)	16 (21.6%)	
- Due to sepsis/MSOF	14 (7%)	0	14 (18.9%)	
- Due to pneumonia (including COVID-19)	4 (2%)	2 (1.5%)	2 (2.7%)	

Footnote: * for patients with multiple complications, the most severe was selected to determine the Clavien–Dindo grade; ^1^ Chi-squared test; ^2^ Fisher’s exact test and Mann–Whitney test.

**Table 4:** Complications that required surgery in the study groups.

Complication Type (n, %)	Total (n = 22)	Elective Group (n = 10)	Emergency Group (n = 12)	*p*-Value
Anastomotic leak/suture dehiscence	6 (3%)	2 (1.5%)	4 (5.4%)	0.04 *
Evisceration	4 (2%)	2 (1.5%)	2 (2.6%)	
Bowel occlusion	8 (4%)	5 (3.9%)	3 (4%)	
Upper gastrointestinal bleeding	2 (1%)	1 (0.8%)	1 (1.3%)	
Colostomy necrosis	1 (0.5%)	0	1 (1.3%)	
Acute cholecystitis	1 (0.5%)	0	1 (1.3%)	

Footnote: *p* value < 0.05 statistically significant; * Fisher’s exact test.

**Table 5:** Multivariate regression model for predicting severe postoperative complications (Clavien–Dindo grade ≥ 3) for patients operated in emergency for complicated colorectal cancer.

Independent Variables	Coefficient	Std. Error	95% CI	P	r_partial_	r_semipartial_
(Constant)	−1.2169	0.3784	−1.9710 to −0.4628	0.0019		
Age	0.01409	0.004634	0.004858 to 0.02333	0.0033	0.3353	0.3021
MLR_PO1	0.1160	0.03613	0.04405 to 0.1880	0.0020	0.3519	0.3191
pTNM_stage	0.1926	0.07300	0.04706 to 0.3380	0.0102	0.2950	0.2620

**Table 6:** Regression equation for sepsis prediction in emergency subgroup.

Independent Variables	Coefficient	Std. Error	95% CI	P	r_partial_	r_semipartial_
(Constant)	−0.08829	0.06783	−0.2235 to 0.04690	0.1972		
NLR_PO1	0.006798	0.001852	0.003107 to 0.01049	0.0005	0.3947	0.3587
COLOSTOMY_ILEOSTOMY	0.2987	0.08261	0.1341 to 0.4634	0.0005	0.3897	0.3534

**Table 7:** Comparative value of systemic inflammatory biomarkers and multivariate model for predicting sepsis.

Variable	AUC	SE ^a^	95% CI ^b^	Sensitivity	Specificity	Cut-Off Value
MREGR_PredSepsisE	0.921	0.0301	0.836 to 0.970	80%	100%	>0.29
NLR_PO1	0.821	0.0518	0.716 to 0.899	88.8%	66.6%	>12.56
PLR_PO1	0.663	0.100	0.545 to 0.767	55.6%	96.7%	>573.3
WBC_I_PO	0.552	0.108	0.434 to 0.666	66.7%	0%	<20
SII_PO_1	0.729	0.0900	0.615 to 0.825	66.7%	90%	>6121.25
MLR_PO1	0.615	0.104	0.496 to 0.724	44.4%	95%	>2

^a^ SE = standard error; ^b^ CI= confidence interval.

**Table 8:** Regression equation for the predictive model for severe postoperative complications in elective patients.

Independent Variables	Coefficient	Std. Error	95% CI	t	P	r_partial_	r_semipartial_
(Constant)	−0.07791	0.05401	−0.1848 to 0.02896	−1.4425	0.1516		
PLR_PO1	0.0003621	0.0001260	0.0001128 to 0.0006115	2.8738	0.0048	0.2462	0.2330
Anemia	0.2021	0.05572	0.09180 to 0.3123	3.6262	0.0004	0.3052	0.2940

## Data Availability

Data are available on requirement due to privacy reasons.

## Supplementary Materials

The following supporting information can be downloaded at: https://www.mdpi.com/article/10.3390/medicina60091481/s1, Supplementary file S1: STROBE Statement—checklist of items that should be included in reports of observational studies.
