# Supplementary material for: The Value of Systemic Inflammatory Indices for Predicting Early Postoperative Complications in Colorectal Cancer
Source: Medicina (Kaunas). 2024 Sep 11;60(9):1481. doi: 10.3390/medicina60091481 (PMC11434509; doi:10.3390/medicina60091481)
Supplement: Supplementary file 1 [file medicina-60-01481-s001.zip › medicina-3203841-supplementary.pdf]

STROBE Statement—checklist of items that should be included in reports of observational studies

	Item No	*Recommendation*	*Page*
Title and abstract	1	(a) Indicate the study's design with a commonly used term in the title or the abstract<br>(b) Provide in the abstract an informative and balanced summary of what was done and what was found	[1]<br>[1]
Introduction<br>*Background/ rationale*	2	Explain the scientific background and rationale for the investigation being reported	[2]
Objectives	3	State specific objectives, including any prespecified hypotheses	[2, 3]
Methods			
Study design	4	Present key elements of study design early in the paper	[3]
Setting	5	Describe the setting, locations, and relevant dates, including periods of recruitment, exposure, follow-up, and data collection	[3]
Participants	6	Give the eligibility criteria and the sources and methods of selection of participants.	[3]
Variables	7	Clearly define all outcomes, exposures, predictors, potential confounders, and effect modifiers.	[3]
Data sources/ measurement	8	For each variable of interest, give sources of data and details of methods of assessment (measurement). Describe comparability of assessment methods if there is more than one group	[3]
Bias	9	Describe any efforts to address potential sources of bias	[3]

Study size	10	Explain how the study size was arrived at	Not applicable
Quantitative variables	11	Explain how quantitative variables were handled in the analyses. If applicable, describe which groupings were chosen and why	[4]
Statistical methods	12	Describe all statistical methods, including those used to control for confounding	[3, 4]
Results			
Participants	13	Report numbers of individuals at each stage of study—eg numbers potentially eligible, examined for eligibility, confirmed eligible, included in the study, completing follow-up, and analyzed	[4]
Descriptive data	14	Give characteristics of study participants (eg demographic, clinical, social) and information on exposures and potential confounders	[4,5]
Outcome data	15	Report numbers of outcome events or summary measures over time	[5]
Main results	16	Give unadjusted estimates and, if applicable, confounder-adjusted estimates and their precision (eg, 95% confidence interval). Make clear which confounders were adjusted for and why they were included	[5, 6, 7, 8, 9, 10, 11, 12]
Other analyses	17	Report other analyses done—eg analyses of subgroups and interactions, and sensitivity analyses	[10,11,12]
Discussion			
Key results	18		[12, 13]

Limitations	19	Summarize key results with reference to study objectives<br>Discuss limitations of the study, taking into account sources of potential bias or imprecision. Discuss both direction and magnitude of any potential bias	[12, 13]
Interpretation	20	Give a cautious overall interpretation of results considering objectives, limitations, multiplicity of analyses, results from similar studies, and other relevant evidence	[13]
Generalizability	21	Discuss the generalizability (external validity) of the study results	[13,14]
Other information			
Funding	22	Give the source of funding and the role of the funders for the present study and, if applicable, for the original study on which the present article is based	[14]
